# Cases and Method of Skin Grafting

**Published:** 1873-10

**Authors:** 


### CASES AND METHOD OF SKIN GRAFTING.

In a recent number of the *British Medical Journal* a report is given, from the Royal Infirmary, of Edinburg, of Mr. J. Bell's method of skin grafting.

In procuring portions of skin for grafting, Mr. Bell takes them from some sound portion of the patient's body, preferably from the arm. A piece of skin is pinched up by a pair of common catch-forceps, and cut off to the required size with a pair of scissors. This piece is divided into smaller pieces about the size of a grain of rice, and is planted among the granulations of the ulcer by means of a probe, one small piece being sufficient for about a square inch of surface. Over each of the grafts is laid a piece of gutta percha tissue, half a square inch in size, previously dipped in some antiseptic solution. The ulcer is then covered by two layers of similar pieces of gutta percha tissue placed on each other in an imbricated manner, and over these a dressing of antiseptic gauze and a bandage. This dressing is not removed for two or three days, when it is replaced as at first. To insure success before grafting, the ulcers should be free from fœtor, and the dressing changed under spray. The advantages of this method of grafting are alleged to be, that no special apparatus is required; that it is extremely simple; and that, by the movement of the pieces of gutta percha on each other, the grafts are protected from all sources of disturbance.

